# Why do intensive care units in countries without board-certified clinical informatics specialists risk falling behind in the Artificial Intelligence era?

**DOI:** 10.62675/2965-2774.20260314

**Published:** 2026-01-28

**Authors:** Rodrigo Octavio Deliberato, Umberto Tachinardi, Eneida A. Mendonça

**Affiliations:** 1 University of Cincinnati College of Medicine Department of Biostatistics, Health Informatics and Data Science Ohio United States Department of Biostatistics, Health Informatics and Data Science, University of Cincinnati College of Medicine - Ohio, United States.; 2 Cincinnati Children's Hospital Medical Center Division of Biomedical Informatics Ohio United States Division of Biomedical Informatics, Cincinnati Children's Hospital Medical Center - Ohio, United States.

## INTRODUCTION

It is well known that global excitement for Artificial Intelligence (AI) has reached an all-time high in healthcare, offering transformative opportunities across clinical decisions, diagnostics, operations, and administrative functions.^(1)^ Intensive care units (ICUs) are especially well-positioned to benefit from AI, as patients are continuously monitored, producing a rich, multimodal stream of data that makes it ideal for real-time decision support, pattern recognition, and precision medicine approaches.^(2,3)^

However, realizing this potential is a complex, multidisciplinary task that spans every component of a product or solution, from needs assessment to development or selection, evaluation, data and systems integration, lifecycle management, and oversight. AI-enabled technologies in the ICU require advanced data pipelines, robust transformations, and strong data and AI governance. Most importantly, their success hinges on the human factor and alignment with existing or purposefully adapted clinical workflows. Together, these demands underscore the essential role of the clinician-informaticist, who bridges and coordinates diverse skills and domains. The lack of formal training for intensive care clinical informaticians is an underappreciated barrier that poses risks for AI implementation in the intensive care setting. To fully benefit from the greater automation and cutting-edge decision support enabled through AI, the ICU community must recognize and address the long-overlooked need for specialized human expertise at the intersection of ICU and informatics; otherwise, they will risk falling behind in the AI era.

Although this viewpoint may appear primarily directed at policymakers, hospital administrators, and information technology (IT) professionals, and may seem disconnected from day-to-day clinical practice, it is essential to recognize that medical liability related to the (mis)use of healthcare AI remains unresolved, raising profound ethical and legal concerns.^(4)^ We propose that this debate has direct implications for all bedside clinicians, patient safety, and the patient-clinician relationship.

## WHY DO INTENSIVE CARE UNITS NEED A CLINICIAN-INFORMATICIAN?

Every day, ICUs around the world generate over 10,000 discrete multimodal data points, encompassing clinical data from electronic health records (EHRs), imaging, laboratory results, multi-omics panels, and second-by-second physiologic waveforms.^(5)^ These data, when uniquely combined with life-threatening physiologic derangements, make the ICU an ideal environment for deriving benefits from AI applications. However, unlike other clinical systems, AI tools have entered healthcare through fragmented, decentralized pathways, inundating clinicians with an excessive number of algorithms and applications, each with its own data modality, interoperability, and technical requirements, as well as safety assessments that require specific risk management frameworks and potential regulatory considerations. However, in contrast to outpatient or office-based settings that allow greater individual autonomy in selecting apps or digital tools, the ICU operates as a complex, highly collaborative environment in which decisions are made in real time by multidisciplinary teams, relying on shared information. In such a setting, individual freedom to choose different tools may not only be ineffective but also undesirable. Still, it could also lead to harm if those tools are not adequately integrated into the broader system.

While practical ICU innovation requires strong multidisciplinary collaboration among intensivists, data scientists, and IT specialists, the clinician-informaticist plays a distinct and complementary role by bridging clinical practice and informatics, thereby ensuring that AI-driven solutions are not only technically sound but also clinically meaningful and safe for patient care. Yet, navigating the technical and environmental complexity of the fast-paced evolution of AI is far from straightforward, and unlocking its value in the ICU requires clinician-informaticians who understand both patient care and system infrastructure.

For example, if a sepsis-prediction model over-alerts in patients with chronic kidney disease, an ICU-embedded clinician-informatician would quickly identify and address the issue, auditing, recalibrating the model, and adjusting alerts to maintain trust before the tool is abandoned. Their role also spans the entire AI lifecycle: from technology selection and integration to validation, monitoring, and decommissioning or retraining. Each phase requires standardized governance that ensures safety, fairness, compliance, and sound clinical judgment, areas where clinician-informaticians are essential.

## CLINICAL INFORMATICS TRAINING: GLOBAL SNAPSHOT AND REMAINING GAPS

For clinician-informaticians to thrive, training should be widely offered, accessible, standardized, and scalable. While clinical informatics is formally recognized in several high-income countries, global standardization is uneven. In the U.S., it became an American Board of Medical Specialties recognized subspecialty in 2013, with more than 60 accredited fellowships^(6)^ offering clear paths to leadership roles like Chief Medical Information Officer,^(7)^ Chief Research Informatics Officer,^(8)^ Chief Health Ai Officer,^(9)^ and Chief Digital Health Officer.^(10,11)^

The U.K. and Canada offer postgraduate informatics education but lack national board certification, which limits professional recognition.^(12)^ Across Europe, few countries offer formal physician certification; most rely on master level programs and support from organizations like the European Federation for Medical Informatics,^(13)^ which do not provide board certification. Brazil also lacks formal recognition by medical boards for clinical informatics. Academic programs offer master's, doctoral, and certificate-level training, primarily in major urban centers. In 2011, the Brazilian Society of Health Informatics introduced a professional certification for healthcare professionals, including physicians. However, national training capacity remains limited, and the field is still not officially recognized by national research funding agencies or the Ministry of Health. In most low- and middle-income countries, there is no formal recognition, with training limited to short courses and on-the-job learning. This global inconsistency leaves many health systems without the expertise to implement AI safely in ICUs.

## CONSEQUENCES FOR INTENSIVE CARE UNIT ARTIFICIAL INTELLIGENCE ADOPTION IN COUNTRIES WITHOUT BOARD-CERTIFIED CLINICAL INFORMATICS

The United States sets the pace in medical AI, hosting most of the training datasets, producing the majority of peer-reviewed literature, and training qualified personnel.^(14,15)^ While intensivists around the world adopt these U.S.-generated insights as end users, many ICUs have yet to invest in building local expertise, as their American counterparts have. Without investing in training a dedicated clinical informatics team, ICUs globally risk falling behind in the AI era, missing the opportunity to build in-house expertise that could guide their priorities, infrastructure development, and digital strategy.

A recent meta-analysis has emphasized this risk analyzing more than 1,263 ICU- based AI studies and reveled that most of the AI applications being developed for ICU focus on predictive analytics such as mortality, length of stay, readmission risk, adverse events, and prognosis models, as well as diagnostic assistance tools, treatment recommendations, intelligent alarm systems, patient education support, and clinical notes assistance. However, the practical implementation of these AI tools remains limited worldwide. The United States stands out as the leading contributor to studies integrating such solutions into the clinical workflow, reflecting its advanced level of maturity in clinical informatics. In contrast, regions with lower levels of clinical informatics, risk significant delays in adopting these valuable, transformative technologies.^(16)^

Another consequence is inefficient or flawed selection and implementation of adequate solutions, leading to increased potential for incidents driven by alert fatigue or faulty alerts, data quality issues that yield incorrect AI-generated insights, and missed opportunities, including the attainment of international accreditations or participation in multinational datasets and clinical trials. To bridge the gap, some ICUs may become overly reliant on vendor-driven recommendations, yielding little to no return on investment for their specific needs and potentially exposing themselves to regulatory challenges and prohibitive costs. Notably, a comprehensive analysis of Food and Drug Administration (FDA)-approved AI applications revealed that fewer than 10% of these approvals included prospective studies for post-market surveillance. That detailed scientific publications accompanied less than 2% of safety and efficacy assessments.^(17)^ This highlights not only the significant risks of deploying such technologies without thorough evaluation and oversight but also underscores the critical role of clinical informatics specialists in ensuring safe and effective integration into healthcare.

Finally, the lack of a formal informatics credential appropriately updated for the AI era limits intensivists’ access to hospital- or system-level leadership roles in health IT, often leaving strategic decisions to non-clinician IT professionals with limited bedside experience. Only a clinician-informatician can bridge clinical needs and technical requirements, ensuring digital tools truly support workflow and patient care.

## RECOMMENDATIONS

While not every intensivist must become a clinical informatics expert, every ICU should ensure access to appropriately trained personnel. At the same time, even in countries where clinical informatics is formally recognized and training programs exist, such specialists may not be consistently available across hospitals, underscoring the need for additional strategies to ensure their effective integration into ICU workflows.

To achieve this, we propose a few potential steps. While it is clear that national boards should recognize clinical informatics as a subspecialty, we acknowledge that this is a political process that may take time. We further argue that both digital health grants and external accreditations, such as those from the Joint Commission, could help accelerate this process if they tied their requirements to the presence of qualified clinical informatics personnel. In the meantime, ICU leaders, aware of the importance of clinical informatics and the risks of operating without it, could allocate internal and external funding for one- to two-year fellowships based on ten core competencies as illustrated in table 1.^(10)^ It is important to note that the majority of clinical informatics training programs and certifications were developed before the widespread adoption of AI and therefore fall short in preparing professionals for today's needs. Beyond covering core competencies, it is essential to encourage additional AI-focused upskilling through Massive Open Online Courses (e.g., edX, Coursera) and institutional partnerships with health centers that are further along in implementing AI tools in the ICU setting, to facilitate and accelerate technical learning.

**Table 1:** Core clinical informatics competencies

Core content area	Key topics
Discipline fundamentals	Fundamental informatics concepts, models, and theories in the discipline of clinical informatics. Understanding of the environment in which they function
1. Health information systems	Electronic medical records/electronic health records, clinical databases, medical imaging systems (PACS), pharmacy order systems, laboratory information systems, telehealth platforms
2. Clinical decision support	Knowledge-based systems, alerting/reminder systems, evidence-based medicine integration, medication safety alerts, workflow integration, and innovation through informatics tools and processes
3. Data governance and analytics	Clinical analytics, big data in healthcare, statistical legacy (t-tests, regression, machine learning, Artificial Intelligence), data acquisition, management, visualization. Data governance structures, policies, and processes
4. Interoperability and standards	Health Level Seven International, Fast Healthcare Interoperability Resources, digital imaging and communications in medicine, Integrating the Healthcare Enterprise Profiles, Data Exchanges (HIE), Application Programming Interface Design, standards compliance
5. Privacy and security	Health Insurance Portability and Accountability Act, Health Information Trust Alliance, General Data protection Law, General Data Protection Regulation, data encryption, role-based access controls, incidental data discovery, audit logs, cybersecurity best practices
6. Legal, ethical and policy	Medical Licensing, Patient Consent (in digitized care), Data Ownership, Artificial Intelligence regulation, risk mitigation, licensure, cultural competency
7. Health information technology project management	Software development lifecycle), change management, user requirements gathering, agile/scrum, risk management, budgeting, stakeholder alignment
8. Usability and human factors	User-centered design, heuristic evaluation, cognitive engineering, workflow alignments, error prevention, anthropometrics, integrated formative testing
9. Organizational change management	Stakeholder analysis, workflow mapping, training programs, adoption metrics, physician burnout prevention, gap analysis, return on investment calculation
10. Research and innovation	Electronic health record-based research, Artificial Intelligence/machine learning integration, quality improvement, pilot studies, grant writing, dissemination strategies, extensible application markup collaborative tools

Lastly, establishing ICU clinical informatics subcommittees co-chaired by intensivists can generate substantial early value and momentum, even in settings without a dedicated clinical informatics expert or before such expertise is widely available. These subcommittees should also play a central role in participating in or helping create enterprise-level data and AI governance structures, taking a key role in the AI lifecycle, including reviewing and approving initiatives to ensure safe, clinically relevant, and ethically responsible deployment.

Intensivist participation provides a unique clinical perspective in evaluating AI applications, including the types of data used (from less sensitive to highly sensitive patient-health information), the potential severity or risk of the application (low, medium, high), and the level of cognitive support the tool provides (inform, drive, decide). Their involvement helps ensure that AI integration aligns with patient safety priorities and ICU workflows, while highlighting the critical need for clinical informatics training.

## CONCLUSIONS

Although intensive care units generate some of the richest clinical data, they risk falling behind in the Artificial Intelligence transformation. Strategic investment in clinical informatics expertise is a low-cost, high-impact opportunity to empower intensive care units, reduce vendor dependency, and improve outcomes. The future of intensive care depends not only on technology but on the people who guide its use, and intensivists with clinical informatics and AI-centric skills must be at the center of this transformation.

## Data Availability

The contents are already available.

## References

[1] Howell MD, Corrado GS, DeSalvo KB (2024). Three epochs of Artificial Intelligence in health care. JAMA.

[2] Burki TK (2021). Artificial intelligence hold promise in the ICU. Lancet Respir Med.

[3] Bulgarelli L, Deliberato RO, Johnson AE (2020). Prediction on critically ill patients: the role of "big data". J Crit Care.

[4] Mello MM, Guha N (2024). Understanding liability risk from using health care Artificial Intelligence tools. N Engl J Med.

[5] Sanchez-Pinto LN, Luo Y, Churpek MM (2018). Big data and data science in critical care. Chest.

[6] Detmer DE, Shortliffe EH (2014). Clinical informatics: prospects for a new medical subspecialty. JAMA.

[7] Leviss J, Kremsdorf R, Mohaideen MF (2006). The CMIO—a new leader for health systems. J Am Med Inform Assoc.

[8] Sanchez-Pinto LN, Mosa AS, Fultz-Hollis K, Tachinardi U, Barnett WK, Embi PJ (2017). The Emerging Role of the Chief Research Informatics Officer in Academic Health Centers. Appl Clin Inform.

[9] Beecy AN, Longhurts CA, Singh K, Watcher RM, Murray SG (2024). The Chief Health AI Officer – An Emerging Role for an Emerging Technology. NEJM AI.

[10] Gardner RM, Overhage JM, Steen EB, Munger BS, Holmes JH, Williamson JJ (2009). AMIA Board of Directors. Core content for the subspecialty of clinical informatics. J Am Med Inform Assoc.

[11] Davies A, Mueller J, Hassey A, Moulton G (2021). Development of a core competency framework for clinical informatics. BMJ Health Care Inform.

[12] Jidkov L, Alexander M, Bark P, Williams JG, Kay J, Taylor P (2019). Health informatics competencies in postgraduate medical education and training in the UK: a mixed methods study. BMJ Open.

[13] Roger France FH, Beguin C, Mélot C, Gillet P (2010). Board certified physicians in health informatics a European precedent for professional recognition. Yearb Med Inform.

[14] Celi LA, Cellini J, Charpignon ML, Dee EC, Dernoncourt F, Eber R (2022). for MIT Critical Data. Sources of bias in artificial intelligence that perpetuate healthcare disparities-A global review. PLOS Digit Health.

[15] Alberto IR, Alberto NR, Altinel Y, Blacker S, Binotti WW, Celi LA (2024). A scientometric analysis of fairness in health AI literature. PLOS Glob Public Health.

[16] Berkhout WE, van Wijngaarden JJ, Workum JD, van de Sande D, Hilling DE, Jung C (2025). Operationalization of Artificial Intelligence Applications in the Intensive Care Unit: A Systematic Review. JAMA Netw Open.

[17] Muralidharan V, Adewale BA, Huang CJ, Nta MT, Ademiju PO, Pathmarajah P (2024). As coping review of reporting gaps in FDA-approved AI medical devices. NPJ Digit Med.

